# Serum total testosterone and the prognosis of patients with advanced liver disease: a systemic review and meta-analysis

**DOI:** 10.7717/peerj.20571

**Published:** 2026-01-13

**Authors:** Xiao-Yu Zhang, Heng-Han Xu, Jian-Hui Ma, Yang Liu, Han Li, Hao-Qian Tan, Jun-Ying Liu

**Affiliations:** 1 Zhoukou Central Hospital Affiliated to Xinxiang Medical University, Zhoukou, China; 2 Zhoukou Central Hospital, Zhoukou, China

**Keywords:** Advanced liver disease, Androgen, Meta-analysis, Mortality, Testosterone

## Abstract

**Background:**

Advanced liver disease (ALD) is associated with significant morbidity and mortality worldwide. Emerging evidence suggests that sex hormonal imbalances, particularly low serum total testosterone (TT) levels, may influence the prognosis of patients with ALD. This meta-analysis aimed to evaluate the association between serum TT levels and the prognosis of patients with ALD.

**Methods:**

Comprehensive searches of PubMed, Embase, and Web of Science were performed from the inception of the searched databases up to November 13, 2025, to identify observational studies assessing the association between serum TT levels and the risk of all-cause mortality or liver transplant (LT) among patients with ALD. Pooled risk ratios (RRs) with 95% confidence intervals (CIs) were calculated using a random-effects model to account for the potential influence of heterogeneity. This systematic review was registered in PROSPERO (CRD42024578870).

**Results:**

Eight cohort studies encompassing 1,989 patients were included in the analysis. Findings demonstrated that low serum TT levels were significantly associated with an increased risk of all-cause mortality or LT during follow-up (RR: 1.87, 95% CI [1.57–2.23], *p* < 0.001; *I*^2^ = 0%). Sensitivity analyses, including those limited to male patients, confirmed the stability of these results. Subgroup analyses revealed consistent associations across various study designs, geographic regions, patient ages, TT cutoff values, follow-up durations, and quality assessments (*p* for subgroup difference, all > 0.05).

**Conclusion:**

Lower serum TT levels are significantly associated with a higher risk of all-cause death or LT in patients with ALD, indicating their potential utility as prognostic biomarkers.

## Introduction

Advanced liver disease (ALD), encompassing conditions such as liver cirrhosis, severe hepatitis, and liver failure *etc.*, remains a significant global health challenge (Huang et al., 2023; Orman et al., 2021; Ufere, 2021). The burden of ALD is reflected in its high morbidity and mortality rates, with complications like hepatic encephalopathy, and variceal bleeding contributing to poor clinical outcomes (Garcia-Martinez, Diaz-Ruiz & Poncela, 2022; Karcz et al., 2012; Tellez & Guerrero, 2022). Despite advances in medical care, the prognosis of patients with ALD remains poor, particularly for those who develop complications requiring liver transplantation (LT) (Peng et al., 2019). Identifying reliable prognostic factors that can predict poor outcomes in these patients is crucial for optimizing management strategies, refining patient stratification, and guiding therapeutic interventions.

Sex hormone imbalances, especially a reduction in serum total testosterone (TT) levels, are common in patients with ALD (Neong, Billington & Congly, 2019; Sinclair et al., 2015; Xu et al., 2022). The liver plays a pivotal role in sex hormone metabolism, and hepatic dysfunction in ALD can lead to significant alterations in hormone levels (Kur et al., 2020). Low TT is particularly prevalent in ALD, with studies reporting that up to 90% of male patients with cirrhosis exhibit testosterone deficiency (Naveen Kumar Rajput & Shekhar, 2023; Vaishnav et al., 2023; Zang et al., 2022). The mechanisms underlying this phenomenon include downregulation of the hypothalamic-pituitary-testicular axis (HPT) in the context of systemic inflammation and the chronic disease state of ALD (Van Thiel et al., 1990), as well as increased aromatization of testosterone to estradiol in the context of liver dysfunction (Papadimitriou et al., 2024). There is a growing body of evidence suggesting that low TT levels may be linked to poor clinical outcomes in ALD (Sinclair et al., 2015). The rationale for this association is grounded in the role of testosterone in maintaining muscle mass, immune function, and overall metabolic health—factors that are critically compromised in ALD (Chrysavgis et al., 2024). Therefore, testosterone deficiency may exacerbate sarcopenia, frailty, and immune dysregulation, leading to an increased risk of mortality and the need for LT (Yang & Kim, 2021).

However, the current literature is characterized by small sample sizes, variability in patient characteristics, differences in study design, and inconsistent cutoff values for defining low TT, which limits the generalizability of individual study findings (Apostolov et al., 2023; Grossmann et al., 2012; Huang et al., 2021; Mallepally et al., 2019; Paternostro et al., 2019; Sinclair et al., 2016b; Sinclair et al., 2016a; Sun et al., 2021). Moreover, a comprehensive meta-analysis to summarize the existing evidence of the relationship between low TT and prognosis in patients with ALD is still needed. Accordingly, this meta-analysis aims to systematically evaluate the association between serum TT levels and the risk of all-cause mortality or LT in patients with ALD, thereby providing critical insights into the prognostic value of TT in this vulnerable population.

## Materials and Methods

The meta-analysis followed the *Preferred Reporting Items for Systematic reviews and Meta-Analyses (PRISMA) 2020* statement (Page et al., 2021) and the *Cochrane Handbook for Systematic Reviews and Meta-analyses* (Higgins et al., 2021) guidelines, which covers the process of study design, data extraction, data analysis, and findings interpretation. These methods were further refined by following the predefined protocols required for the study’s registration with the International Prospective Register of Systematic Reviews (PROSPERO; registration number CRD42024578870).

### Database search

We performed a comprehensive literature search across PubMed, Embase, and Web of Science to identify relevant studies, employing a combination of predefined search terms, including: (“testosterone” OR “androgen” OR “dihydrotestosterone” OR “DHT”) AND (“advanced liver disease” OR “end-stage liver disease” OR “end stage liver disease” OR “cirrhosis” OR “cirrhotic” OR “liver fibrosis” OR “hepatitis” OR “liver failure” OR “hepatic failure”) AND (“death” OR “deaths” OR “mortality” OR “survival” OR “transplantation” OR “transplant” OR “transplant-free survival” OR “TFS” OR “prognosis”). The search was limited to research involving human subjects, and we only included studies published in English. Additionally, we manually reviewed the references of relevant original and review articles to identify further pertinent studies. The literature was assessed from the inception of the searched databases up to November 13, 2025. The complete search strategy for each included database is detailed in File S1.

### Study eligibility criteria

Eligibility criteria for study selection were established based on the Population, Intervention, Comparison, Outcome, and Study design (PICOS) framework.

P (Population): Patients with confirmed diagnosis of ALD, such as severe hepatitis, cirrhosis, and acute or chronic liver failure.

I (Exposure): Patients with a low serum level of total testosterone (TT), defined by the cutoff values used in the primary studies.

C (Comparison): Patients with normal (or high) serum TT.

O (Outcome): Incidence of the composite outcome of all-cause mortality or liver transplant (LT), compared between patients with and without a lower serum level of TT at enrollment.

S (Study design): Observational designs with longitudinal follow-up, encompassing cohort studies, nested case-control studies, and post-hoc analyses derived from clinical trials.

Studies were excluded if they were reviews, editorials, meta-analyses, preclinical investigations, or cross-sectional in design. We also excluded studies involving populations with conditions other than advanced liver disease, those that did not assess serum total testosterone as the exposure of interest, and those that failed to report outcomes related to all-cause mortality or LT.

### Study quality assessment and data collection

The literature search and study selection were independently conducted by Xiao-Yu Zhang and Heng-Han Xu. Any discrepancies between the reviewers were resolved through discussion, and unresolved disagreements were adjudicated by the corresponding author, Jun-Ying Liu. The methodological quality of the included studies was appraised using the Newcastle–Ottawa Scale (NOS) (Wells et al., 2010). This scale evaluates three domains: cohort selection, control of confounding factors, and assessment of outcomes. Scores range from 1 to 9, with higher scores indicating better methodological quality. Extracted data included study characteristics (first author, publication year, study location, and design), participant information (clinical diagnosis, sample size, age, sex distribution, and baseline Model for End-stage Liver Disease [MELD] scores), timing of serum TT assessment, methods for measuring TT, cutoff values used to define low TT levels, duration of follow-up, number of deaths or LT during follow-up, and covariates accounted for in the analysis of the relationship between serum TT and clinical outcomes in patients with advanced liver disease.

### Statistical analysis

We assessed the association between serum TT levels and the risk of all-cause mortality or LT in patients with advanced liver disease using risk ratios (RRs) and corresponding 95% CIs, comparing individuals with low *versus* normal TT at baseline. When studies reported odds ratios (ORs) instead of RRs, we converted them using the following formula: RR = OR/([1 − pRef] + [pRef × OR]), where pRef represents the prevalence of the outcome in the reference group (patients without a low TT) (Zhang & Yu, 1998). RRs and their standard errors were derived from reported 95% CIs or *p*-values and log-transformed to stabilize variance. Heterogeneity among studies was evaluated using Cochrane’s Q test and the *I*^2^ statistic (Higgins & Thompson, 2002), with *I*^2^ > 50% representing substantial heterogeneity. A random-effects model was employed to pool the effect estimates, incorporating between-study variability to account for potential heterogeneity (Higgins et al., 2021). To evaluate the robustness of the findings, sensitivity analyses were conducted by sequentially excluding individual studies from the meta-analysis. An additional sensitivity analysis was limited to studies that exclusively enrolled male participants. Prespecified subgroup analyses were also undertaken to investigate whether factors such as study design, geographic region, average age of participants, threshold values for defining low TT, follow-up duration, and study quality (as indicated by NOS) modified the association between TT and mortality in patients with advanced liver disease. In addition, subgroup analyses according to the patient diagnosis and methods for measuring TT were also performed. For continuous variables, subgroup cutoffs were based on their median values. The overall certainty of the evidence was assessed using the Grading of Recommendations Assessment, Development, and Evaluation (GRADE) framework, which considers five domains: risk of bias (within and across studies), inconsistency, indirectness, imprecision, and potential for publication bias (Guyatt et al., 2011). Potential publication bias was assessed through visual inspection of funnel plot symmetry and further examined using Egger’s regression test (Egger et al., 1997). All statistical analyses were conducted using RevMan (version 5.1, Cochrane Collaboration, Oxford, UK) and Stata (version 12.0, StataCorp, College Station, TX, USA).

## Results

### Study inclusion

**Figure 1:**
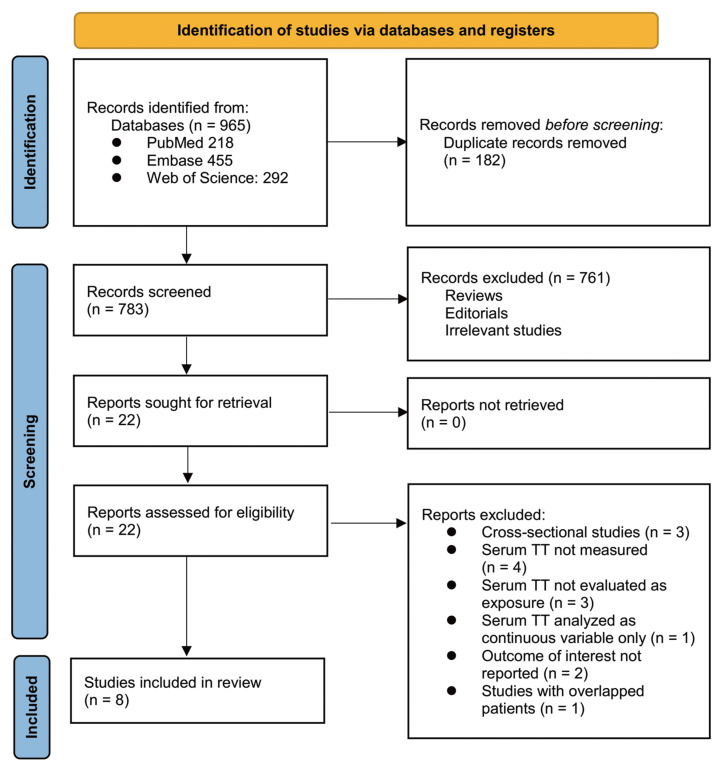
PRISMA flowchart of database search and study inclusion.

The study selection process is summarized in Fig. 1. A total of 965 potentially relevant articles were retrieved from the three databases. After removing 182 duplicates, 783 records remained for title and abstract screening. Of these, 761 were excluded for not meeting the eligibility criteria or lacking relevance to the objectives of the meta-analysis. The full texts of the remaining 22 articles were then assessed independently by two reviewers, leading to the exclusion of 14 studies for reasons outlined in Fig. 1. Ultimately, eight cohort studies met the inclusion criteria and were incorporated into the quantitative synthesis (Apostolov et al., 2023; Grossmann et al., 2012; Huang et al., 2021; Mallepally et al., 2019; Paternostro et al., 2019; Sinclair et al., 2016b; Sinclair et al., 2016a; Sun et al., 2021).

### Overview of the study characteristics

Table 1 shows the summarized characteristics of the available studies included in the meta-analysis. Overall, four prospective cohort studies (Huang et al., 2021; Paternostro et al., 2019; Sinclair et al., 2016b; Sun et al., 2021) and four retrospective studies (Apostolov et al., 2023; Grossmann et al., 2012; Mallepally et al., 2019; Sinclair et al., 2016a) were included in the meta-analysis. These studies were published from 2012 to 2023, and were conducted in Spain, Australia, the United States, Austria, and China. One study included patients with ALD on the LT list (Grossmann et al., 2012), five studies included patients with liver cirrhosis (Apostolov et al., 2023; Mallepally et al., 2019; Paternostro et al., 2019; Sinclair et al., 2016b; Sinclair et al., 2016a), and another two studies included patients with hepatitis B virus (HBV)—related acute-on-chronic liver failure (ACLF) (Huang et al., 2021; Sun et al., 2021). Overall, 1,989 patients with ALD were included in this meta-analysis. The mean ages of the included patients were 45 years to 61 years. Seven of the included studies enrolled male patients only (Apostolov et al., 2023; Grossmann et al., 2012; Huang et al., 2021; Mallepally et al., 2019; Paternostro et al., 2019; Sinclair et al., 2016b; Sinclair et al., 2016a), whereas in another study, male patients accounted for 87% of the enrolled patients (Sun et al., 2021). The mean MELD score varied from 10.0 to 21.3 among the included patients. Serum TT was measured at the time of patient enrollment. The methods for measuring TT were chemiluminescence immunoassay (CLIA) in three studies (Grossmann et al., 2012; Sinclair et al., 2016b; Sinclair et al., 2016a), electrochemiluminescence immunoassay (ECLIA) in three studies (Apostolov et al., 2023; Paternostro et al., 2019; Sun et al., 2021), and chemi-bioluminescent immunoassay (CBLIA) in one study (Huang et al., 2021). The method for measuring TT was not reported in one study (Mallepally et al., 2019). The cutoff values for defining a low TT ranged from 4.0 nM to 21.6 nM. The mean follow-up durations were 1 month to 13 months, and a total of 990 (49.8%) patients died or received LT during the follow-up period. Potential confounding factors, such as age, sex, and MELD scores, were adjusted to a varying degree in the multivariate analytic model when the association between serum TT and the clinical outcome of patients with ALD was evaluated. The NOS scores of the included studies were seven to nine, suggesting an overall good study quality (Table 2).

**Table 1:** Summarized study characteristics of the included studies.

Study	Location	Study design	Diagnosis	Sample size	Mean age (years)	Men (%)	Mean MELD scores	Timing for measuring TT	Methods for measuring TT	Cutoff for low TT	Mean follow-up duration (months)	No. of patients who died or received LT	Variables adjusted
Grossmann et al. (2012)	Australia	RC	Men with ALD on LT list	171	53	100	16	At enrollment	CLIA	5 nM	8	119	Age, sodium, and MELD score
Sinclair et al. (2016a)	Australia	RC	Men with decompensated liver cirrhosis for LT evaluation	145	54	100	17	At enrollment	CLIA	8 nM	8.3	135	Age, MELD score, and muscle mass
Sinclair et al. (2016b)	Australia	PC	Men with cirrhosis	268	55	100	10	At enrollment	CLIA	8.3 nM	12	50	Age and MELD score
Mallepally et al. (2019)	USA	RC	Men with cirrhosis	257	61	100	NR	At enrollment	NR	Median (NR)	NR	156	Age, and MELD-Na score
Paternostro et al. (2019)	Austria	PC	Men with cirrhosis	114	55	100	13.6	At enrollment	ECLIA	21.6 nM	13	70	Age, CPS, HCC, MELD score, SCr, albumin, and presence of ascites
Huang et al. (2021)	China	PC	Men with HBV related ACLF	160	45	100	NR	At enrollment	CBLIA	4.8 nM	3	55	Age, BMI, INR, BUN, TB, DB, WBC, PLT, TC and TP
Sun et al. (2021)	China	PC	Patients with HBV related ACLF	108	46.2	87	21.3	At enrollment	ECLIA	4 nM	1	24	Age, sex, comorbidities, treatments, WBC, TB, ALT, AST, albumin, INR, sodium, and SCr
Apostolov et al. (2023)	Australia	RC	Men with cirrhosis	766	56	100	14	At enrollment	ECLIA	12 nM	12	381	Age, MELD score, and CPS

**Notes.**

ACLF acute-on-chronic liver failure ALD advanced liver disease ALT alanine aminotransferase AST aspartate aminotransferase BMI body mass index BUN blood urea nitrogen CBLIA chemi-bioluminescent immunoassay CLIA chemiluminescence immunoassay CPS Child–Pugh Score DB direct bilirubin ECLIA electrochemiluminescence immunoassay HBV hepatitis virus B HCC hepatocellular carcinoma INR international normalized ratio LT liver transplant MELD Model for End-stage Liver Disease NR not reported PC prospective cohort PLT platelet RC retrospective cohort SCr serum creatinine TB total bilirubin TC total cholesterol TP total protein TT total testosterone WBC white blood cell

**Table 2:** Study quality evaluation *via* the Newcastle-Ottawa Scale.

Study	Representativeness of the exposed cohort	Selection of the non-exposed cohort	Ascertainment of exposure	Outcome not present at baseline	Control for age and sex	Control for other confounding factors	Assessment of outcome	Enough long follow-up duration	Adequacy of follow-up of cohorts	Total
Grossmann et al. (2012)	0	1	1	1	1	1	1	0	1	7
Sinclair et al. (2016a)	0	1	1	1	1	1	1	0	1	7
Sinclair et al. (2016b)	1	1	1	1	1	1	1	1	1	9
Mallepally et al. (2019)	1	1	0	1	1	1	1	0	1	7
Paternostro et al. (2019)	1	1	1	1	1	1	0	1	1	8
Huang et al. (2021)	1	1	1	1	1	1	1	0	1	8
Sun et al. (2021)	1	1	1	1	1	1	1	0	1	8
Apostolov et al. (2023)	0	1	1	1	1	1	1	1	1	8

### Results of the meta-analysis and sensitivity analysis

**Figure 2:**
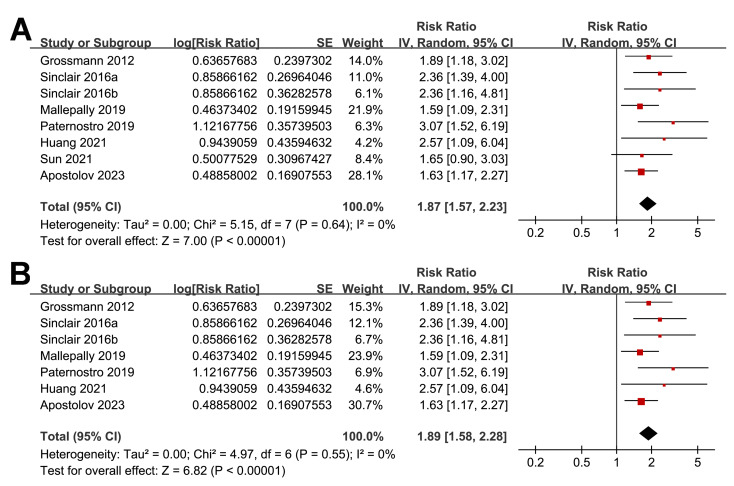
Forest plots for the meta-analysis of the association between a low serum TT and the risk of all-cause mortality or LT in patients with ALD. (A) The overall meta-analysis; (B) The sensitivity analysis limited to studies with male patients only. ALD, advanced liver disease; LT, liver transplant; TT, total testosterone. Studies: Apostolov et al., 2023; Grossmann et al., 2012; Huang et al., 2021; Mallepally et al., 2019; Paternostro et al., 2019; Sinclair et al., 2016b; Sinclair et al., 2016a; Sun et al., 2021.

Overall, the pooled results of the eight included studies showed that a lower serum TT at enrollment was significantly related to an increased risk of all-cause mortality or LT in patients with ALD during follow-up (RR: 1.87, 95% CI [1.57–2.23], *p* < 0.001; *I*^2^ = 0%; Fig. 2A), with moderate certainty of evidence (Table 3). The sensitivity analyses were performed by excluding one dataset at a time but did not significantly change the results (RR: 1.81–1.98, *p* all < 0.05). In particular, a further sensitivity analysis limited to the seven studies including male patients only (Apostolov et al., 2023; Grossmann et al., 2012; Huang et al., 2021; Mallepally et al., 2019; Paternostro et al., 2019; Sinclair et al., 2016b; Sinclair et al., 2016a) also showed similar results (RR: 1.89, 95% CI [1.58–2.28], *p* < 0.001; *I*^2^ = 0%; Fig. 2B).

**Table 3:** Summary of findings: Association between serum total testosterone levels and prognosis in patients with advanced liver disease—impact of low serum testosterone at enrollment on all-cause mortality and liver transplant outcomes.

Results	Effect (95% CI)	N (n)	Certainty of the evidence (GRADE)	Comments
Association between serum TT at baseline and the risk of all-cause mortality or LT in patients with ALD	RR 1.87 (1.57–2.23)	1,989 (8)	Moderate^a^	Low TT at enrollment is associated with an increased risk of all-cause mortality or LT in ALD patients. The certainty is moderate due to the observational nature of included studies.

**Notes.**

a Downgraded one point due to the observational nature of the included studies, which limits causal inference despite adjustments for confounders.

ALD advanced liver disease CI confidence interval GRADE Grading of Recommendations Assessment, Development, and Evaluation LT liver transplant n total number of studies N total number of participants RR risk ratio TT total testosterone

GRADE Working Group Grades of Evidence: High certainty: Our confidence is very high that the true effect lies close to the estimated effect; Moderate certainty: Our confidence is moderate in the estimated effect. The true effect is likely to be close to the estimate, but there is a possibility it is substantially different; Low certainty: Our confidence in the estimated effect is limited. The true effect may be substantially different.

Very low certainty: Our confidence is very low in the estimated effect. The true effect is likely to be substantially different.

### Results of the subgroup analyses

**Figure 3:**
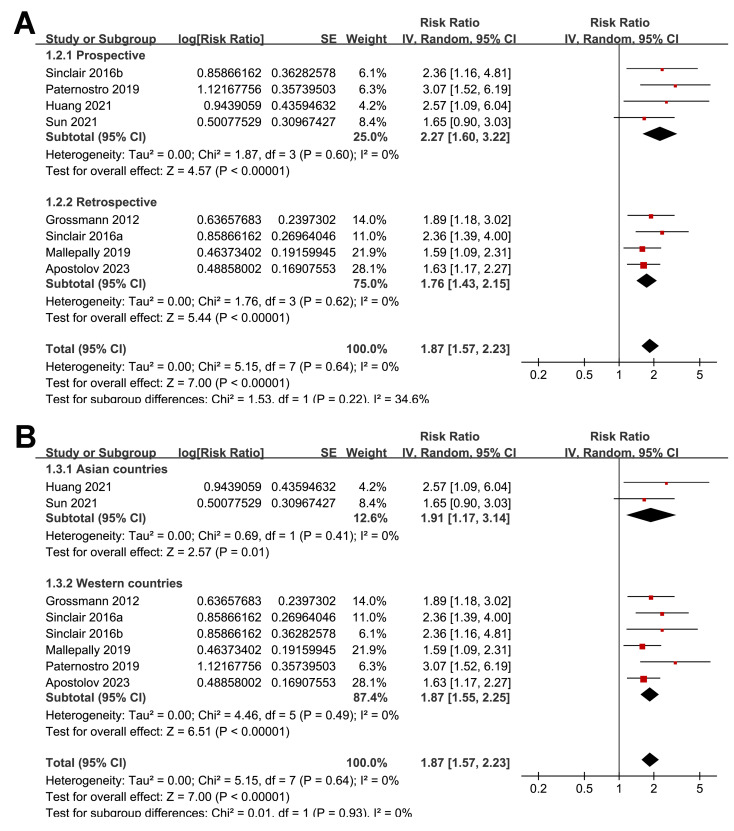
Forest plots for the subgroup analyses of the association between a low serum TT and the risk of all-cause mortality or LT in patients with ALD. (A) The subgroup analysis according to study design; (B) the subgroup analysis according to study country. ALD, advanced liver disease; LT, liver transplant; TT, total testosterone. Studies: Apostolov et al., 2023; Grossmann et al., 2012; Huang et al., 2021; Mallepally et al., 2019; Paternostro et al., 2019; Sinclair et al., 2016b; Sinclair et al., 2016a; Sun et al., 2021.

**Figure 4:**
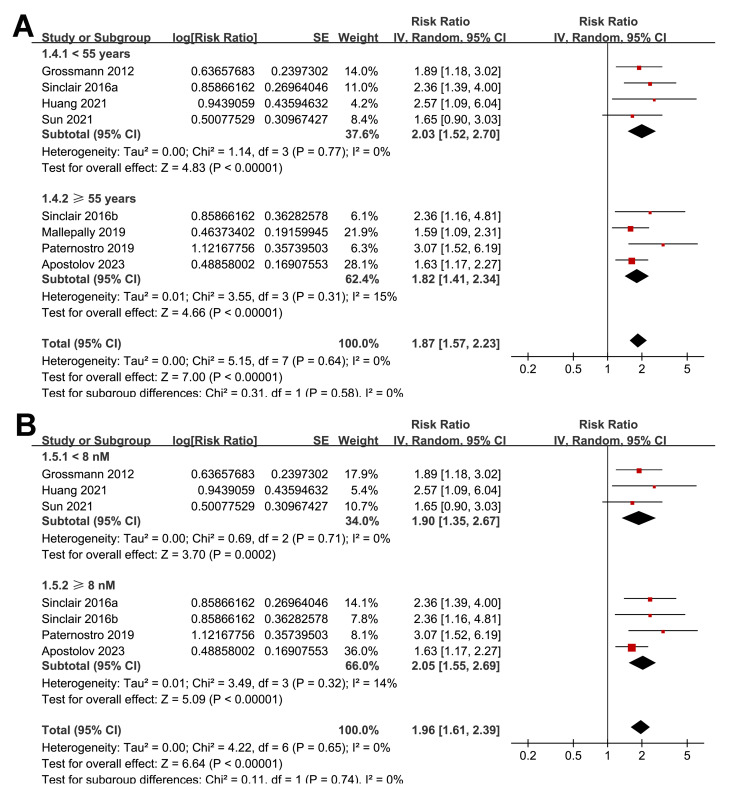
Forest plots for the subgroup analyses of the association between a low serum TT and the risk of all-cause mortality or LT in patients with ALD. (A) The subgroup analysis according to the mean age of the patients; (B) the subgroup analysis according to the cutoff values for defining a low TT. ALD, advanced liver disease; LT, liver transplant; TT, total testosterone. Studies: Apostolov et al., 2023; Grossmann et al., 2012; Huang et al., 2021; Mallepally et al., 2019; Paternostro et al., 2019; Sinclair et al., 2016b; Sinclair et al., 2016a; Sun et al., 2021.

**Figure 5:**
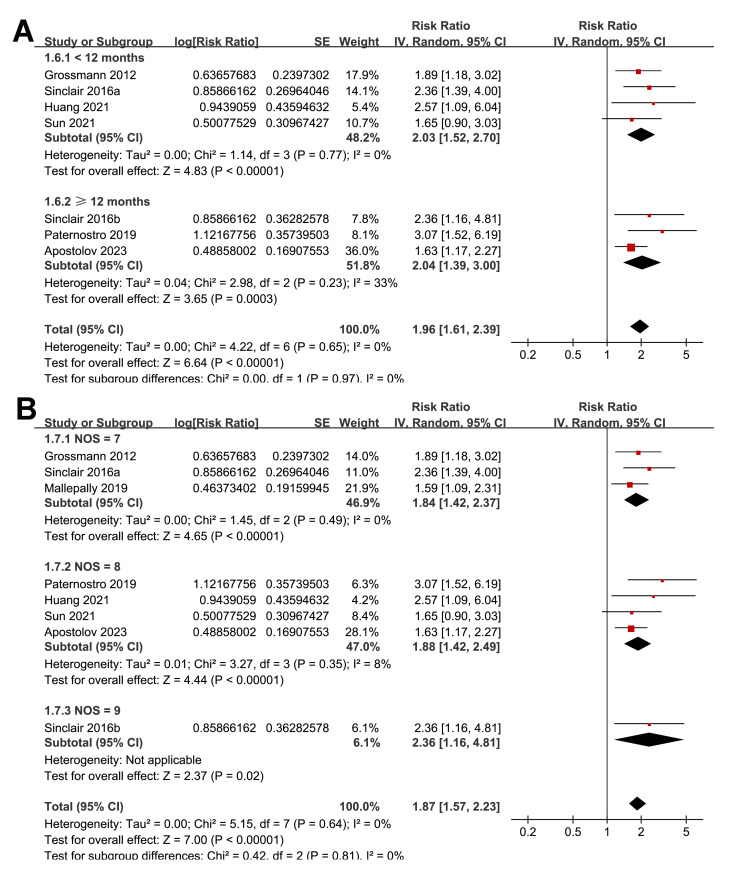
Forest plots for the subgroup analyses of the association between a low serum TT and the risk of all-cause mortality or LT in patients with ALD. (A) The subgroup analysis according to the mean follow-up durations; (B) the subgroup analysis according to the NOS scores. ALD, advanced liver disease; LT, liver transplant; TT, total testosterone; NOS, Newcastle–Ottawa Scale. Studies: Apostolov et al., 2023; Grossmann et al., 2012; Huang et al., 2021; Mallepally et al., 2019; Paternostro et al., 2019; Sinclair et al., 2016b; Sinclair et al., 2016a; Sun et al., 2021.

**Figure 6:**
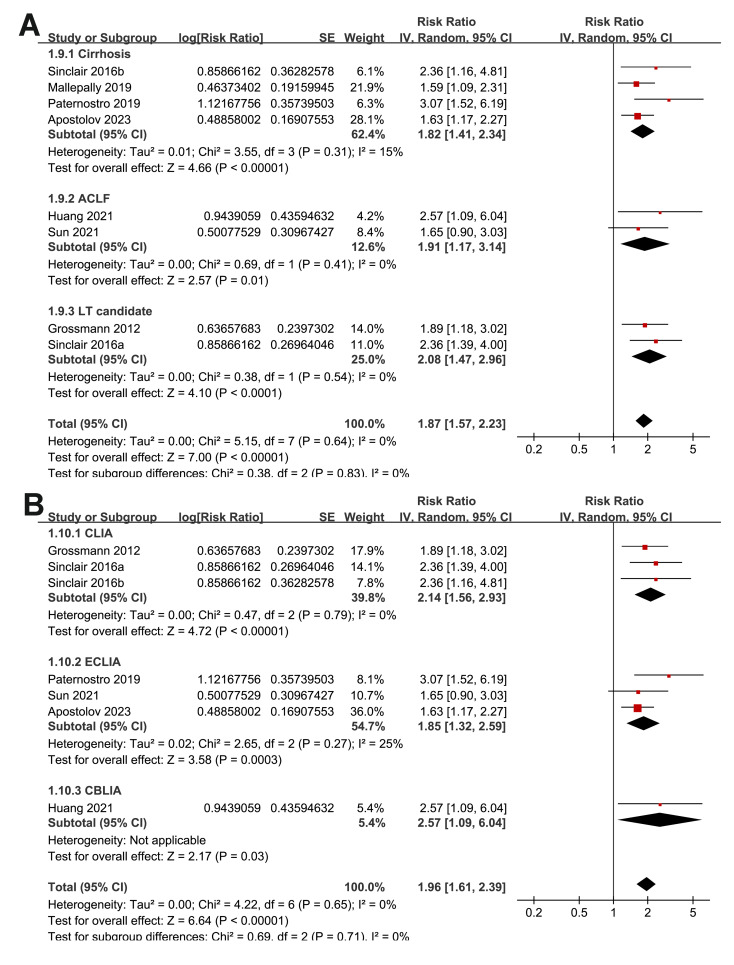
Forest plots for the subgroup analyses of the association between a low serum TT and the risk of all-cause mortality or LT in patients with ALD. (A) The subgroup analysis according to the diagnosis of the patients; (B) the subgroup analysis according to the methods for measuring TT. ALD, advanced liver disease; LT, liver transplant; TT, total testosterone; ACLF, acute-on-chronic liver failure; CBLIA, chemi-bioluminescent immunoassay; CLIA, chemiluminescence immunoassay; ECLIA, electrochemiluminescence immunoassay. Studies: Apostolov et al., 2023; Grossmann et al., 2012; Huang et al., 2021; Mallepally et al., 2019; Paternostro et al., 2019; Sinclair et al., 2016b; Sinclair et al., 2016a; Sun et al., 2021.

The subgroup analyses indicated that the association between a low serum TT and the risk of all-cause mortality or LT in patients with ALD was consistent among prospective and retrospective cohorts (RR: 2.27 *versus* 1.76, *p* for subgroup difference = 0.22; Fig. 3A); in studies from Asian and western countries (RR: 1.91 *versus* 1.87, *p* for subgroup difference = 0.93; Fig. 3B); in patients with a mean age < 55 and ≥ 55 years old (RR: 2.03 *versus* 1.82, *p* for subgroup difference = 0.58; Fig. 4A); in studies with cutoff values for defining a low TT < 8 and ≥ 8 nM (RR: 1.90 *versus* 2.05, *p* for subgroup difference = 0.74; Fig. 4B); in studies with a mean follow-up duration of <12 and ≥ 12 months (RR: 2.03 *versus* 2.04, *p* for subgroup difference = 0.97; Fig. 5A); and in studies with NOS scores of seven, eight, and nine (RR: 1.84 *versus* 1.88 and 2.36, *p* for subgroup difference = 0.81; Fig. 5B). In addition, the results were consistent for patients with cirrhosis, ACLF and as candidates for LT (RR: 1.82, 1.91, and 2.08, *p* for subgroup difference = 0.81; Fig. 6A), and for studies with TT measured with CLIA, ECLIA, and CBLIA (RR: 2.15, 1.85, and 2.57, *p* for subgroup difference = 0.71; Fig. 6B).

### Publication bias

**Figure 7:**
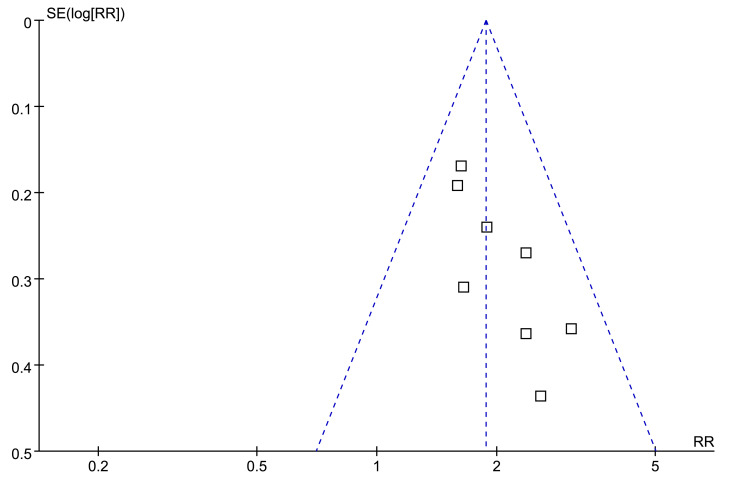
Funnel plots for meta-analysis of the association between a low serum TT and the risk of all-cause mortality or LT in patients with ALD. ALD, advanced liver disease; LT, liver transplant; TT, total testosterone.

Visual examination of the funnel plots for the association between low serum TT and the risk of all-cause mortality or LT revealed a symmetrical distribution, suggesting minimal publication bias (Fig. 7). This observation was further supported by Egger’s regression test, which yielded a non-significant result (*p* = 0.38), indicating a low probability of publication bias.

## Discussion

This meta-analysis provides compelling evidence that low serum TT levels may be significantly associated with an increased risk of all-cause mortality or the need for LT in patients with ALD. The pooled analysis reveals a nearly two-fold increase in the risk of adverse outcomes among those with lower TT levels at enrollment. These findings may support the potential role of TT as a prognostic biomarker for ALD and suggest that hormonal evaluation could be integrated into routine clinical assessments for patients with this condition.

The underlying mechanisms linking low TT levels to worse outcomes in ALD are likely multifactorial, involving both direct and indirect effects on various physiological systems. Testosterone is a key regulator of anabolic processes, including muscle protein synthesis and maintenance of lean body mass (Jain et al., 2022). In patients with ALD, the downregulation of the HPT axis combined with systemic inflammation and nutritional deficiencies, can lead to significant reductions in TT levels (Jain et al., 2022; Van Thiel et al., 1990). This hormonal imbalance exacerbates sarcopenia, a condition characterized by a loss of muscle mass and function, which is common in cirrhosis and strongly associated with poor clinical outcomes, including increased mortality and higher rates of LT (Ebadi et al., 2019; Leunis et al., 2023).

At the molecular level, testosterone influences several signaling pathways that are critical for maintaining metabolic and immune homeostasis (Shepherd et al., 2020). One of the key pathways affected by testosterone is the PI3K/AKT/mTOR pathway, which promotes protein synthesis and inhibits protein degradation in muscle cells (Basualto-Alarcon et al., 2013). In the context of low testosterone, the suppression of this pathway can lead to muscle wasting, contributing to the frailty and diminished physical reserve observed in patients with ALD (Saad et al., 2017). This frailty, in turn, increases vulnerability to complications such as infections, hepatic encephalopathy, and variceal bleeding, all of which can precipitate the need for LT or lead to death (Padhi et al., 2024; Xie, Jing & Yang, 2024; Yuan et al., 2024). In addition to its anabolic effects, testosterone has immunomodulatory properties that may be particularly relevant in the setting of ALD. Testosterone modulates the production of pro-inflammatory cytokines, such as tumor necrosis factor-alpha (TNF-α) and interleukin-6 (IL-6) (Mohamad et al., 2019), which are elevated in chronic liver disease and contribute to liver injury and fibrosis progression (Martinez-Esparza et al., 2015). Low TT levels may exacerbate this pro-inflammatory milieu, further driving liver damage and impairing the body’s ability to mount an effective immune response to infections (Nakashima et al., 2017). This could explain the increased susceptibility to sepsis and other severe infections seen in hypogonadal patients (Hackett et al., 2023). Studies are still warranted to determine the key molecular pathways underlying the association between a low TT and the poor prognosis of these patients.

A sensitivity analysis of studies including predominantly male patients confirmed the robustness of the findings, with little variation in the risk estimates when individual studies were excluded. This consistency suggests that the association between low TT and adverse outcomes is not driven by any single study and reflects a true underlying relationship. However, the exclusion of studies involving female patients limits the generalizability of the results to women (Mazer, 2002), who also experience significant hormonal imbalances in the context of liver disease. The lack of data on women in the included studies is a significant limitation, as the pathophysiology of hormonal changes and their impact on prognosis may differ between genders. More studies are needed to determine the influence of TT on the prognosis of women with ALD. Subgroup analyses further bolstered the findings by demonstrating consistent associations across various study characteristics, including study design, geographic region, patient age, TT cutoff values, study quality scores, patient diagnosis and methods for measuring TT. These consistent findings across different subgroups suggest that the prognostic value of TT is robust and applicable across diverse clinical settings and patient populations.

To the best of our knowledge, this meta-analysis is likely the first comprehensive review to systematically evaluate the association between serum TT levels and clinical outcomes in patients with ALD. The inclusion of only cohort studies, all of which conducted multivariate analyses to adjust for potential confounders, enhances the credibility of the findings. This approach minimizes the risk of bias and ensures that the observed associations are not merely the result of confounding factors, such as age, sex, or severity of liver disease, which are known to influence both TT levels and clinical outcomes in ALD. Additionally, the large pooled sample size increases the statistical power and precision of the estimates, providing a more definitive assessment of the prognostic significance of low TT in this vulnerable population. However, some limitations of this meta-analysis should be acknowledged. The exclusion of female patients from most of the included studies means that the implications for women with ALD remain unclear. Given the differences in sex hormone physiology between men and women, it is possible that the relationship between TT and clinical outcomes may differ in female patients; this should be explored in future studies. Furthermore, the variability in the TT cutoff values used across studies could introduce heterogeneity, although this was addressed through subgroup analyses. In addition, there is heterogeneity of the included patient population, which ranged from cirrhosis to ACLF and LT candidates under the broad category of ALD. While these stages differ in certain pathophysiological aspects, they share common features, such as advanced hepatic dysfunction, systemic inflammation, hormonal dysregulation, and a high risk of adverse outcomes. Moreover, our subgroup analysis stratified by diagnosis (Fig. 6A) showed consistent associations across all disease stages with minimal heterogeneity, suggesting that the main findings were robust despite this limitation. Another limitation is the observational nature of the included studies, which precludes the ability to infer causality definitively. While the use of multivariate analyses helped to control for confounding factors, residual confounding cannot be entirely ruled out. Additionally, none of the included studies involved interventional treatments, and the treatment details were generally not reported, which prevented us from assessing the potential influence of specific therapeutic interventions on patient outcomes.

Recent evidence provides additional context for interpreting the prognostic value of testosterone in ALD. A 2025 observational study evaluating TT as a continuous variable reported that total testosterone did not independently predict mortality in the overall cohort after multivariable adjustment (Adamantou et al., 2025). However, TT level was negatively associated with mortality risk in men-specific models, suggesting that sex-specific hormonal dynamics may influence its prognostic impact (Adamantou et al., 2025). These findings align with our pooled results showing a consistent inverse association between testosterone and adverse outcomes, while also highlighting that the strength of this association may vary depending on analytic approaches and population characteristics. Importantly, moving beyond observational associations, a recent target-trial emulation (Tapper, Chen & Parikh, 2025) demonstrated that testosterone replacement therapy in older men with newly diagnosed cirrhosis and hypogonadism was associated with lower mortality and fewer decompensation events, including reduced ascites requiring paracentesis and variceal hemorrhage, without increasing hepatocellular carcinoma risk. Together, these emerging data complement our findings and indicate that low TT may represent not only a biomarker of advanced physiological deterioration but also a potentially modifiable risk factor that warrants further prospective investigation.

From a clinical perspective, the findings of this meta-analysis highlight the importance of considering hormonal evaluation in the management of patients with ALD. Assessing TT levels could help identify high-risk patients who may benefit from more intensive monitoring and therapeutic interventions. For instance, patients with low TT may require closer surveillance for complications such as infections or hepatic decompensation and may be prioritized for LT evaluation. Additionally, the potential role of testosterone replacement therapy (TRT) in improving outcomes for patients with ALD warrants further investigation. While TRT is not currently a standard treatment for testosterone deficiency in ALD due to concerns about potential adverse effects, such as worsening fluid retention or exacerbating liver inflammation (Wang & Swerdloff, 2022), small studies have suggested that TRT may have beneficial effects on muscle mass, strength, and quality of life in hypogonadal men with cirrhosis (Sinclair et al., 2016). Randomized controlled trials are needed to determine whether TRT can safely and effectively improve clinical outcomes in this population. Accordingly, in terms of future research directions, studies that include female patients are urgently needed to understand the role of TT and other sex hormones in determining prognosis in women with ALD. Additionally, research should focus on elucidating the specific molecular mechanisms through which low TT contributes to poor outcomes in ALD, with an emphasis on identifying potential therapeutic targets. Understanding the interplay between testosterone, systemic inflammation, and liver fibrosis could lead to the development of novel treatments that address both the hormonal and hepatic aspects of ALD. Furthermore, prospective studies with standardized definitions of low TT and longer follow-up periods are needed to validate the findings of this meta-analysis and to explore the long-term implications of low TT on LT-free survival in ALD.

## Conclusions

In conclusion, this meta-analysis suggests that low serum TT levels may be significantly associated with an increased risk of all-cause mortality and the need for LT in patients with ALD, a finding which was primarily driven by studies with male patients. These findings suggest that TT may serve as a valuable prognostic biomarker in this population, offering potential opportunities for risk stratification and targeted therapeutic interventions. Given the high morbidity and mortality associated with ALD, integrating hormonal evaluation into clinical practice could provide critical insights into patient prognosis and inform treatment decisions. Further research is needed to explore the potential benefits of testosterone replacement and to verify these findings in female patients with ALD.

##  Supplemental Information

10.7717/peerj.20571/supp-1 Supplemental Information 1 The raw data extracted from the cited literature

10.7717/peerj.20571/supp-2 Supplemental Information 2 Search strategy for each database

10.7717/peerj.20571/supp-3 Supplemental Information 3 PRISMA checklist

10.7717/peerj.20571/supp-4 Supplemental Information 4 Rationale

## Additional Information and Declarations

### Data Availability

The following information was supplied regarding data availability: The raw data are available in the Supplemental File.
