# Supplementary material for: Serum total testosterone and the prognosis of patients with advanced liver disease: a systemic review and meta-analysis
Source: PeerJ. 2026 Jan 13;14:e20571. doi: 10.7717/peerj.20571 (PMC12810399; doi:10.7717/peerj.20571)
Supplement: Supplemental Information 2 [file peerj-14-20571-s002.docx]

**Search strategy for each database**

**PubMed**

(“Testosterone”[MeSH] OR “Androgens”[MeSH] OR testosterone OR androgen OR dihydrotestosterone OR DHT) AND (“Liver Cirrhosis”[MeSH] OR “Liver Failure”[MeSH] OR “Liver Diseases”[MeSH] OR “Hepatitis”[MeSH] OR “Fibrosis”[MeSH] OR “End-Stage Liver Disease” OR “Cirrhosis” OR “Cirrhotic” OR “Liver Fibrosis” OR “Liver Failure” OR “Hepatic Failure” OR “Advanced Liver Disease”) AND (“Prognosis”[MeSH] OR “Survival Analysis”[MeSH] OR “Mortality”[MeSH] OR “Transplantation”[MeSH] OR “Death” OR “Deaths” OR “Transplant” OR “Transplant-free survival” OR “TFS”)

**Embase**

('testosterone'/exp OR 'androgen'/exp OR testosterone OR androgen OR dihydrotestosterone OR DHT) AND ('liver cirrhosis'/exp OR 'hepatic failure'/exp OR 'liver disease'/exp OR 'hepatitis'/exp OR 'liver fibrosis'/exp OR 'end stage liver disease' OR 'advanced liver disease' OR cirrhosis OR cirrhotic OR 'liver failure' OR 'hepatic failure') AND ('prognosis'/exp OR 'survival'/exp OR 'mortality'/exp OR 'transplantation'/exp OR 'death' OR 'deaths' OR 'transplant' OR 'transplant-free survival' OR 'TFS')

**Web of Science**

TS=(testosterone OR androgen OR dihydrotestosterone OR DHT) AND TS=(“advanced liver disease” OR “end-stage liver disease” OR “end stage liver disease” OR cirrhosis OR cirrhotic OR “liver fibrosis” OR hepatitis OR “liver failure” OR “hepatic failure”) AND TS=(prognosis OR survival OR mortality OR death OR deaths OR transplant OR transplantation OR “transplant-free survival” OR TFS)
