# Supplementary material for: Serum total testosterone and the prognosis of patients with advanced liver disease: a systemic review and meta-analysis
Source: PeerJ. 2026 Jan 13;14:e20571. doi: 10.7717/peerj.20571 (PMC12810399; doi:10.7717/peerj.20571)
Supplement: Supplemental Information 4 [file peerj-14-20571-s004.docx]

**Systematic Review and/or Meta-Analysis Rationale**

**1. The rationale for conducting the systematic review/meta-analysis**

Advanced liver disease (ALD) is associated with high rates of morbidity and mortality. As liver function declines, endocrine disturbances, particularly testosterone deficiency, become increasingly prevalent. Emerging research has suggested a possible association between low serum total testosterone (TT) levels and poor outcomes such as death or the need for liver transplantation. However, these studies vary widely in design, patient population, testosterone cutoff values, and outcome definitions. To date, no comprehensive meta-analysis has synthesized the available evidence regarding this association. Therefore, this meta-analysis was conducted to evaluate the relationship between baseline serum TT levels and the prognosis of patients with ALD. The study aims to clarify whether TT can serve as a prognostic biomarker for identifying patients at higher risk of adverse outcomes.

**2. The contribution that it makes to knowledge in light of previously published related reports, including other meta-analyses and systematic reviews**

While several observational studies have explored the prognostic role of testosterone in liver disease, findings remain inconsistent and fragmented. Previous reports have not systematically evaluated or quantified the risk of all-cause mortality or liver transplantation in relation to TT levels in ALD patients. This meta-analysis contributes novel insight by pooling data from multiple cohort studies, assessing study quality, performing subgroup and sensitivity analyses, and evaluating the certainty of evidence. This review is also the first to provide a structured synthesis of how TT correlates with outcomes in ALD, potentially informing future risk stratification models and identifying targets for hormonal evaluation and intervention. It also highlights the limitations of existing literature and the need for further research, especially among female patients.
